# Rapid Molecular Diagnosis of Tuberculosis and Its Resistance to Rifampicin and Isoniazid with Automated MDR/MTB ELITe MGB^®^ Assay

**DOI:** 10.3390/antibiotics10070797

**Published:** 2021-06-30

**Authors:** Vichita Ok, Alexandra Aubry, Florence Morel, Isabelle Bonnet, Jérôme Robert, Wladimir Sougakoff

**Affiliations:** 1 Centre d’Immunologie et des Maladies Infectieuses, Sorbonne Université, INSERM, U1135, Cimi-Paris, F-75013 Paris, France; vichita.ok@aphp.fr (V.O.); alexandra.aubry@sorbonne-universite.fr (A.A.); florence.morel2@aphp.fr (F.M.); isabelle.bonnet2@aphp.fr (I.B.); jerome.robert@aphp.fr (J.R.); 2 AP-HP (Assistance Publique Hôpitaux de Paris), Centre National de Référence des Mycobactéries et de la Résistance des Mycobactéries aux Antituberculeux, Laboratoire de Bactériologie-Hygiène, Groupe Hospitalier Universitaire Sorbonne Université, Hôpital Pitié-Salpêtrière, F-75013 Paris, France

**Keywords:** tuberculosis, multi-drug resistance, rapid molecular diagnosis

## Abstract

The MDR/MTB ELITe MGB^®^ kit (ELITech) carried on the ELITe InGenius^®^ platform is a new real-time PCR assay allowing automated extraction and detection of DNA of the *Mycobacterium tuberculosis* complex (MTB) and mutations in the *rpoB* and *katG* genes and *inhA* promoter region (pro-*inhA*) associated to resistance to rifampicin and isoniazid, the two markers of multidrug-resistant TB (MDR). We assessed the performances of the test on a collection of strains (*n* = 54) and a set of clinical samples (*n* = 242) from routine practice, comparatively to TB diagnosis and genotypic drug susceptibility testing (gDST) as references. Regarding the 242 clinical samples, the sensitivity and specificity of MTB detection by ELITe were 90.9% and 97.5%, respectively. For the detection of resistance-conferring mutations on positive clinical samples, we observed perfect agreement with gDST for *katG* and pro-*inhA* (κ = 1.0) and two discordant results for *rpoB* (κ = 0.82). Considering the 54 cultured strains, very good agreement with gDST was observed for the detection of the 25 distinct mutations in *rpoB*, *katG*, and pro-*inhA*, (κ = 0.95, 0.88, and 0.95, respectively). In conclusion, the automated MDR/MTB ELITe MGB^®^ assay shows great promise and appears to be a valuable tool for rapid detection of pre-MDR- and MDR-TB directly from clinical specimens.

## 1. Introduction

Antibiotic resistance is a major threat to tuberculosis (TB) control. Efficient patient care needs early and accurate detection of *Mycobacterium tuberculosis* complex (MTB) and identification of resistance to rifampicin (RIF-R) and isoniazid (INH-R) [1]. The World Health Organization (WHO)-endorsed Xpert^®^ MTB/RIF assay (Cepheid, Sunnyvale, CA, USA) has the advantage of being fully automated but provides limited information as it can only detect *rpoB* mutations involved in RIF-R but not INH-R, which can significantly impact first-line regimen success. Molecular-based assays have been developed to detect specific drug resistance-encoding mutations in MTB [2,3,4]. For instance, GenoType MTBDR*plus* (Hain Lifescience, Nehren, Germany) assay is recommended by the WHO for direct testing of smear-positive specimens and MTB isolates. It permits the detection within 48 h of the most frequent mutations in *rpoB* and in *katG* genes, and *inhA* promoter (pro-*inhA*), which cause RIF-R and INH-R, respectively [5,6,7]. This line-probe assay is not automated and is time-consuming. Other “real-time” PCR-based assays detecting resistance to both RIF and INH have been developed, such as Fluorotype^®^ MTBDR VER 2.0 (Hain Lifescience, Nehren, Germany), which detects mutations in the same three targets as GenoType MTBDR*plus* for both RIF and INH resistance, and is additionally automated with a testing capacity of 95 samples per run. However, automation does not comprise DNA extraction [8].

MDR/MTB ELITe MGB^®^ Kit on the ELITe InGenius^®^ platform (ELITechGroup Molecular Diagnostics, Bothell, WA, USA) is the first multiplex real-time PCR assay designed for automated DNA extraction and simultaneous detection of the DNA of MTB and of the main mutations responsible for resistance to RIF and INH in clinical specimens. Automation has the advantage of obtaining results in 3 h, with all steps included from sample to results, thus reducing time-to-result and making it possible to initiate early adapted treatment, especially in case of MDR-TB. The assay was first evaluated by Bisognin et al. but on frozen samples spiked with mutated strains [9]. Recently, a second study by Hodille et al. was performed retrospectively on clinical samples (*n* = 54) and strains (*n* = 25) [10]. The present evaluation of the assay aims to provide its diagnostic accuracy directly on 242 specimens collected in prospective clinical routine practice and, in a second part, retrospectively on 54 previously characterized strains from our French National Reference Centre of Mycobacteria (NRC MyRMA, Paris, France) collection, representing a wide range of mutations (25 distinct mutations in total).

## 2. Results

### 2.1. Performances of MDR/MTB ELITe MGB^®^ Assay on Routine Clinical Specimens

The first part of the study aimed to assess the performances of MDR/MTB ELITe MGB^®^ assay as a routine diagnostic test. The assay was performed prospectively on 247 consecutive clinical specimens sent to our laboratory for diagnosis of TB. DNA extraction and amplification were performed directly from the clinical sample on the automated ELITe InGenius^®^ system. Five smear-negative bronchial aspirates were excluded because their results on the ELITe assay were invalid owing to the presence of inhibitors, leaving 242 samples included for comparison in our study.

Out of the 242 specimens, TB diagnosis was confirmed for 44 of them and was excluded for the remaining 198 samples. Among the 44 positives, 40 were ELITe positive for MTB while 4 remained negative (Table 1). The 4 latter, for which the culture was positive, were negative on smear examination and also using another molecular assay (Fluorotype^®^ MTB, Hain). Among the 198 TB negatives, 193 were detected as negative by ELITe while 5 were discordant ELITe positives. Apparent sensitivity and specificity were respectively 90.9% and 97.5% (Table 1).

For the detection of resistance mutations in *rpoB*, *katG*, and pro-*inhA*, ELITe InGenius^®^ software is set up to interpret results only if Ct ≤ 31. Among the 40 concordant ELITe positives displayed on Table 1, ELITe InGenius^®^ was able to interpret resistance results for 26 specimens of Ct ≤ 31, while no valid result was proposed by the InGenius^®^ system for the other 14 specimens for which Ct > 31. Nevertheless, for such low positives, ELITe InGenius^®^ offers the possibility of manual analysis of the curves by the operator. In our study, 6 out of the 14 InGenius^®^-uninterpretable results (all RIF-S and INH-S) could thus be interpreted based on direct analysis of the curves (see ^6^ in Table 2).

For each of these six assays, 100% concordance was observed with gDST for the three targets *rpoB*, *katG*, and *inhA* promoter. It has to be noted that the smear examination was negative for three of them, showed <1 acid-fast bacteria (AFB) for two samples, and 1–9 AFB for the last one. The eight remaining InGenius^®^-uninterpretable results (including two MDR-TB and six RIF-S and INH-S, Table 2) could not be analyzed by the operator either. Six of them were smear negative and one displayed <1 AFB (no smear microscopy was performed for the last one owing to insufficient quantity). Considering the thirty-two results which could be interpreted by either InGenius^®^ (*n* = 26) or the operator (*n* = 6), predictions of resistance by ELITe assay and by gDST perfectly matched for *katG* and pro-*inhA*, with kappa values of 1.0 for both targets (Table 2). Regarding the detection of resistance-conferring mutations in *rpoB*, two discrepancies were observed. First, a rare mutation Asn438Asp was detected by Sanger sequencing, which is not covered by MDR/MTB ELITe MGB^®^ (see ^2^ on Table 2). As this mutation was finally proven not to be linked to RIF resistance (the corresponding strain was RIF-S by phenotypic DST), the non-detection of Asn438Asp by ELITe was finally regarded as a true negative/susceptible result. In another sample (see ^3^ on Table 2), the ELITe resistant/gDST wild-type result was due to the detection of a very weak signal yet below the threshold in the amplification curve of a probe by ELITe, considered as an absence of amplification, hence initially interpreted as resistant by ELITe InGenius^®^. The result was finally considered a false positive. Before interpreting the data, the sensitivity and specificity values found for *rpoB* were 85.7% and 96%, respectively, with a kappa value of 0.82 (Table 2). After interpretation, we determined for *rpoB* sensitivity and specificity values of 85.7 and 100%, respectively, and agreement of ELITe assay with gDST was very good, with a kappa value of 0.90 (Table 2). Because the resistant cases in our set of clinical isolates were due to only two distinct mutations in *rpoB* (Ser450Leu (*n* = 5), His445Asp (*n* = 1)), one in *katG* (Ser315Thr, *n* = 5), and one in pro-*inhA* (−15C > T, *n* = 5), we undertook an evaluation of the ELITe assay on a second set of previously characterized isolates in order to widen the range of mutations tested in these three targets.

### 2.2. Retrospective Study: Performances of MDR/MTB ELITe MGB^®^ Assay in Detecting Resistance Mutations in rpoB, katG, and Pro-inhA on Well-Defined Isolates

In this second part of the study, we aimed to assess the performances of MDR/MTB ELITe MGB^®^ assay in detecting resistance mutations in *rpoB*, *katG*, and pro-*inhA* from 54 isolates from our NRC MyRMA collection. The DNAs extracted by thermal lysis from these isolates were tested with MDR/MTB ELITe MGB^®^ assay using the “only PCR” function of the ELITe InGenius^®^ platform. The DNAs were from 40 MDR strains, 2 mono-RIF resistant strains, 2 mono-INH resistant strains, and 10 strains susceptible to both INH and RIF. The 44 resistant strains represented in total 17 distinct amino acid substitutions in *rpoB*, 5 in *katG*, and 3 mutations in the *inhA* promoter region (Table 3).

The performances in detecting resistance mutations in comparison with gDST are presented in Table 4. None of the 12 strains wild-type for *rpoB* had any *rpoB* mutation detected by ELITe (Table 4). Among the 42 strains mutated in *rpoB*, 41 resistance mutations were detected in *rpoB* by ELITe. The single discordant result was due to an infrequent *rpoB* genetic variant, Val251Phe (Table 3 and ^1^ on Table 4), which was not detected by ELITe since the region covered by the assay is limited to the mutations affecting codons 429 to 452 (the mutation Val251Phe was actually detected after *rpoB* sequencing). Sensitivity and specificity were 97.6% and 100.0%, respectively. Excellent concordance with gDST was observed with a kappa value of 0.95 (Table 4).

Regarding the *katG* gene, the 17 wild-type isolates were well-detected as susceptible by ELITe assay (Table 4). Among the 37 mutant strains, ELITe detected all the mutations appearing at codon 315, namely the frequent Ser315Thr (*n* = 33) but also Ser315Gly (*n* = 1, Table 3 and see ^2^ on Table 4). The latter was detected on a strain which was INH-S on phenotypical DST and was finally considered as a false positive ELITe result. The three remaining discordant ELITe susceptible/gDST mutated results (see ^3^ in Table 4) were due to infrequent mutations located outside the codon 315 region screened by the assay. The first one was Ser140Gly which was not associated to resistance (phenotypically INH-S, Table 3). Therefore, it was interpreted as a true negative result. The other two mutations, Arg249Pro and Trp689Gly, identified on resistant strains, were regarded as two false negative ELITe results. Sensitivity and specificity of ELITe in detecting resistance mutations in *katG* gene were 91.8% and 100% before interpretation, and 94.3% and 94.7% after interpretation, respectively, with a κ = 0.88 showing very good agreement.

Finally, considering the *inhA* promoter region, the single discordant result between ELITe assay and gDST was due to the mutation −60C > G (Table 3 and see ^4^ on Table 4), which is not covered by the ELITe assay. This mutation was identified by Sanger sequencing on an INH-R strain, which presents no other identified mutation. The assay showed a sensitivity and a specificity of 93.3% and 100%, respectively. Total agreement for the detection of resistance mutations in pro-*inhA* was excellent, with a kappa coefficient of 0.95.

## 3. Discussion

In this study performed on 242 routinely collected specimens, MDR/MTB ELITe MGB^®^ assay yielded very good results in diagnosing TB, with a sensitivity and a specificity of 90.9% and 97.5%, respectively. The performance parameters of ELITe assay were significantly higher than those of microscopic examination (sensitivity of 69.0% and specificity of 92.3%), as shown in Table 1. Among the ELITe positives, 14 were smear negatives, with 9 confirmed TB (see ^1^ on Table 1). Interestingly, the ELITe assay seems sensitive enough to detect pauci-bacillary TB. Sensitivity was comparable to that published by Bisognin et al. [9], and higher than that observed by Hodille et al, in routine clinical context in our study. Considering the ELITe negatives, 15 were smear-positive, among which 13 were due to the non-tuberculosis mycobacteria *M. intracellulare* (*n* = 3), *M. avium* (*n* = 5), *M. fortuitum* (*n* = 1), *M. chimaera* (*n* = 2), *M. abscessus* susp. *bolletii* (*n* = 1), and *M. genavense* (*n* = 1), and 2 were false-positive microscopic examinations likely due to *Corynebacterium* spp., indicating very high specificity of ELITe assay, which was also observed by Bisognin et al. (99.23%) and Hodille et al. (100%) [9,10]. Among the nine discordant results, four were false-negative ELITe results obtained from four culture-diagnosed TB with negative microscopic examinations owing to their low bacillary load in the specimens. Notably, these four negative specimens were also negative by Hain FluoroType^®^ MTB, suggesting that molecular diagnostics seem to not be sensitive enough in such smear-negative specimens. On the other hand, five out of the nine discordant results were discordant ELITe positive ones. The five false-positive results, for which no diagnosis of TB had been established, corresponded to ELITe amplification curves showing very late positive and doubtful signals, for which either artefacts or contamination could not be excluded. These observations should warn about InGenius^®^ interpretation issues for very weak positives.

Recently, new rapid tests have been developed, such as Xpert^®^ MTB/RIF Ultra, for which a gain in sensitivity has been reported (88–95.7%), yet raising a specificity issue (58.6–96.7%) [11,12]. Comparatively, the ELITe assay has the advantage of offering high sensitivity while keeping the highest specificity, as it was also recently reported by Bisognin et al. [9]. Hodille et al. found a lower sensitivity on their 54 selected samples, when compared to culture. It has to be noted that clinical specimen processing makes it inappropriate to declare a sensitivity of 100% in detecting RIF and INH resistance, while it was calculated based on only six samples for both molecules [10]. In our study, we tested prospectively as in routine diagnosis 242 samples, among which 44 were confirmed diagnosed TB.

Another advantage of the ELITe assay is that the ELITe InGenius^®^ apparatus allows the recovery of the extracted DNAs for further molecular tests, such as the identification of associated resistance markers (in particular to second-line drugs) and the phylogenotyping allowing to trace rapidly possible routes of transmission of MDR-TB.

Regarding the detection of resistance mutations, MDR/MTB ELITe MGB^®^ assay has been designed to provide results similar to the GenoType MTBDR*plus* assay which targets the same regions in *rpoB*, *katG*, and pro-*inhA*. With respect to the five discordant ELITe susceptible/gDST-mutated results shown on Table 4, the mutations Val251Phe on *rpoB* and Ser140Gly, Arg249Pro, and Trp689Gly on *katG* are not covered by ELITe. Like MTBDR*plus*, the MDR/MTB ELITe assay misses about 7% of the *katG* mutations outside the “315” target [13]. Notably, ELITe was able to detect the mutation Ser315Gly, to which no resistance was associated. It is important to note here that in comparison with the MTBDR*plus* line probe assay, ELITe cannot specify the most frequent mutations but only predicts if a mutation is present in each of the three targets. Therefore, one could wonder about the consequences of silent mutations on the ELITe performances. Even if they could in practice impact the results of the ELITe analysis, silent mutations are scarcely observed, and none was detected in our study. Nevertheless, this point should be assessed with larger sampling in the future. ELITe assay is robust regarding *rpoB* and pro-*inhA* since most associated resistance mutations are located in the regions targeted by the assay. It appeared less robust for *katG* owing to the more diversified mutations outside the tested region, of which the impact on resistance to INH is less predictable.

Our study has two limitations: (i), the size of our samples which has to be enlarged to confirm our results, and (ii) the limited diversity in tested specimens. In fact, our study was performed mostly on pulmonary samples but included only few extra-pulmonary specimens. However, in our study, the assay was evaluated in laboratory routine conditions, unlike the first evaluations of the ELITe assay recently published by Bisognin et al. and Hodille et al. [9,10], which were performed on frozen specimens. In the first evaluation, all the positive specimens were drug-susceptible and wild-type for the three molecular targets *rpoB*, *katG*, and *inhA* promoter, and detection of resistance was assessed on samples spiked with three mutated strains containing only mutations Ser450Leu on *rpoB*, Ser315Thr on *katG*, and −15C > G on *inhA* promoter. As for the second study by Hodille et al., only the mutations on the strains were described. Considering the three targets, only nine mutations were tested, namely Ser450Leu, His445Tyr, and Leu452Pro on *rpoB* gene, Ser315Thr, Δ1–492, Gln88Pro, and Leu343STOP on *katG*, and −15*fabG1* and −17*fabG1* on pro-*inhA* [10]. The large range of mutations tested in our study (25 distinct mutations from clinical isolates, representing frequently observed resistance patterns) provides sufficient diversity to reinforce our confidence in the potential of the ELITe assay to be an efficient routine tool for diagnosing MDR-TB.

## 4. Materials and Methods

### 4.1. Clinical Specimen Processing

Two hundred and forty-seven consecutive routine clinical specimens were collected from 5 November 2019 to 28 August 2020 from liquefied sputa (*n* = 41), bronchial aspirates (*n* = 148), bronchoalveolar lavages (*n* = 12), gastric aspirations (*n* = 5), biopsies (*n* = 18), cerebrospinal fluid (*n* = 7), lymph nodes (*n* = 5), abscesses/pus (*n* = 6), urine (*n* = 1), and other cavitary fluids (*n* = 4), from patients for which TB was suspected. Samples were processed inside a level-III biosafety cabinet. For every specimen, culture on Löwenstein-Jensen (LJ) medium was performed and a slide was prepared and stained with auramine for microscopic examination, following routine procedure in our laboratory [14].

### 4.2. DNAs Extracted from Strains

Fifty-four DNAs from our NRC MyRMA collection were tested. These DNAs were selected in order to cover a wide set of mutations in genes conferring resistance to RIF and INH. These strains belong to the lineages Beijing (*n* = 17), Delhi-CAS (*n* = 2), Ghana (*n* = 3), Haarlem (*n* = 3), H1 (*n* = 1), LAM (*n* = 8), NEW-1 (*n* = 1), S (*n* = 3), Uganda-1/T2 (*n* = 1), T1 (*n* = 2), Ural (*n* = 1), and uncharacterized strains (*n* = 12). DNAs were extracted from isolated strains by thermal lysis, as previously described [15].

### 4.3. Genotypic DST

Detection of resistance mutations in the three targets (*rpoB*, *katG*, and pro-*inhA*) by the new assay was compared with genotypic DST (gDST) as reference, which included identification of mutations in the three targets using GenoType MTBDR*plus* and Sanger sequencing [15] plus Deeplex^®^ Myc-TB (GenoScreen, Lille, France) when required, following the manufacturers’ instructions.

### 4.4. MDR/MTB ELITe MGB^®^ Kit on ELITe InGenius^®^ System (ELITechGroup Molecular Diagnostics, Bothell, WA, USA)

The MDR/MTB ELITe MGB^®^ assay consists of a fully automated multiplex real-time PCR assay designed for the simultaneous detection of the DNA of *M. tuberculosis* and of resistance to rifampicin and isoniazid. Based on the use of ELITe MGB^®^ probes, MTB Complex is detected by targeting the repeated sequence IS*6110*, rifampicin resistance by analyzing the so-called Rifampicin Resistance Determining Region (RRDR) in *rpoB*, and isoniazid resistance by analyzing *katG* at the level of codon 315 and *inhA* at the level of its promoter region. The entire ELITe assay is performed on the ELITe InGenius platform, an automated integrated system for extraction, amplification, detection, and results interpretation.

Specimens were first decontaminated using NAC-PAC^®^ decontamination and digestion system (AlphaTec^TM^), following the manufacturer’s instructions and inactivated using thermal shock (25 min at 95 °C, 1 h at −20 °C, 10 min at 95 °C, and 10 min at −20 °C). Two hundred microliters of decontaminated and inactivated specimens were processed on the ELITe InGenius^®^ System, following the manufacturer’s instructions. Briefly, DNA extractions were performed from 200 µL of sample in ready-to-use, unitary, cassette-based format extraction cartridges, while real-time multiplexed PCR amplifications were performed in independent unit cartridges for Elite-controlled thermal cyclers. An internal control was included at the extraction step in order to control the absence of PCR inhibitors. Results interpretation and analysis were automatically done by the system. The raw data for the Ct values are given as Supplementary Information (Table S1). The cutoff value used by the InGenius^®^ system to differentiate between positive and negative samples is 45.

### 4.5. Statistical Analysis

Apparent sensitivity and specificity of the new assay were calculated in comparison with confirmed diagnosis of TB following the international guidelines [16] for MTB detection and gDST for the detection of resistance mutations as references. Agreements between MDR/MTB ELITe MGB^®^ assay and reference tests were assessed with Kappa statistics since it is more robust for evaluation of agreement between categorical variables. Cohen’s kappa (κ) values from 0.00 to 0.20 show slight agreement, those from 0.21 to 0.40 indicate fair agreement, those from 0.41 to 0.6 suggest moderate agreement, those from 0.61 to 0.80 show substantial agreement, and those above 0.8 indicate excellent agreement [17]. Statistical analyses were performed using R packages epiR [18] and irr [19] on R software [20].

## 5. Conclusions

As a conclusion, MDR/MTB ELITe MGB^®^ assay combines the abilities to yield early diagnostic results directly from specimens, like Xpert^®^ technology, and to properly detect RIF and INH resistance like GenoType MTBDR*plus* DNA strips. The automation on the ELITe InGenius^®^ Instrument, which integrates DNA extraction, amplification, and result interpretation makes it possible to get results in about 3 h, a feature which not only can bring about early results and early adapted treatment for the patients but is also time-sparing for technicians. However, this technique needs appropriate and dedicated premises, which makes it difficult to use as point-of-care testing. In the present report, we have shown that the ELITe assay has proven to be a reliable test to detect MTB and resistance to first-line drugs RIF and INH in clinical routine practice. Its use directly on primary specimens could be very helpful as an early guide for treatment if the assay is combined with a new next-generation sequencing (NGS)-based targeted deep-sequencing approach such as the Deeplex^®^ Myc-TB assay which allows the detection of resistance to 13 anti-TB drugs [21,22]. Such a strategy represents a very promising breakthrough for early optimal treatment of MDR-TB and XDR-TB patients and is currently under evaluation in our laboratory.

## Figures and Tables

**Table 1:** Performances of MDR/MTB ELITe MGB^®^ assay and microscopic examination for the detection of *M. tuberculosis* complex in specimens in comparison to TB diagnosis.

TB Diagnosis	ELITe MTB (*n* = 242)		Microscopic Examination (*n* = 238)	
	Positive (*n*)	Negative (*n*)	Positive (*n*)	Negative (*n*)
Positive	40 ^1^	4	29	13
Negative	5 ^1^	193 ^2^	15	182
Sensitivity, %	90.9		69.0	
(95% CI)	(78.3–97.5)		(52.9–82.4)	
Specificity, %	97.5		92.3	
(95% CI)	(94.2–99.2)		(87.6–95.7)	
Cohen’s Kappa	0.88		0.60	
(95% CI)	(0.80–0.96)		(0.47–0.74)	

^1^ Including 14 smear-negative (9 TB diagnosis positive and 5 TB diagnosis negative). ^2^ Including 15 smear-positive ELITe negative with 13 NTM and 2 contaminated samples.

**Table 2:** Performances of MDR/MTB ELITe MGB^®^ assay in detecting resistance mutations in *rpoB*, *katG*, and pro-*inhA* on 40 ELITe MTB-positive specimens in comparison with gDST.

gDST	MDR/MTB ELITe					
	Resistant (*n*)	Susceptible (*n*)	Uninterpretable (InGenius^®^ and Operator)	Sensitivity (Interpreted) % (95% CI)	Specificity (Interpreted) % (95% CI)	Cohen’s Kappa (Interpreted) (95% CI)
*rpoB*						
Mutated	6 ^1^	1 ^2^	2	85.7 (85.7)	96.0 (100.0)	0.82 (0.90)
Wild-type	1 ^3^	24 ^6^	6	(42.1–99.7)	(79.6–99.9)	(0.57–1.0)
*katG*						
Mutated	5 ^4^	0	2	100 (100)	100 (100)	1.0 (1.0)
Wild-type	0	27 ^6^	6	(47.8–100)	(87.2–100)	(1.0–1.0)
pro-*inhA*						
Mutated	5 ^5^	0	0	100 (100)	100 (100)	1.0 (1.0)
Wild-type	0	27 ^6^	8	(47.8–100)	(87.2–100)	(1.0–1.0)

^1^ detected mutations: Ser450Leu, His445Asp. ^2^ mutation Asn438Asp on *rpoB* gene, not covered by MDR/MTB ELITe MGB^®^, and not conferring resistance to RIF, finally considered as “true negative/susceptible” result. ^3^ absence of detection of a probe, interpreted as resistant by ELITe InGenius^®^. ^4^ detected mutation: Ser315Thr. ^5^ detected mutation: −15C > T. ^6^ including 6 with Ct > 31 (not interpretable by ELITe InGenius^®^) finally interpreted by manual analysis of the curves by the operator.

**Table 3:** Mutations detected in *rpoB* gene, *katG* gene, and *inhA* promoter on clinical specimens and on isolates from our NRC MyRMA collection and tested by MDR/MTB ELITe MGB^®^ assay, according to resistance patterns.

Resistance Phenotype	Mutation in		
	*rpoB*	*katG*	*inhA* Promoter
MDR	Val251Phe; Gln432Pro; Asp435Val; Asp435Tyr; Asp435Tyr + Ser441Thr; Asp435Tyr + Gln429Arg; Asp435Tyr + Met433Ile; Asp435Glu + His445Asn; His445Cys; His445Asp; His445Leu; His445Arg; His445Tyr; Ser450Leu; Leu452Pro	Ser315Thr Trp689Gly	−15C > T −8T > A
Mono RIF-R	Ser450Leu, His445Tyr	Ser140Gly ^1^	
Mono INH-R	Asn438Asp ^2^	Arg249Pro	−60C > G
Susceptible	Leu430Pro	Ser315Gly ^3^	

^1^ Ser140Gly in *katG*: not conferring resistance to isoniazid. ^2^ Asn438Asp in *rpoB*: not conferring resistance to rifampicin. ^3^ Ser315Gly in *katG*: not conferring resistance to isoniazid.

**Table 4:** Performances of MDR/MTB ELITe MGB^®^ assay in detecting resistance mutations in *rpoB*, *katG*, and pro-*inhA* on strains (*n* = 54) from our NRCMyRMA collection, in comparison with gDST.

gDST	MDR/MTB ELITe Results				
	Resistant (*n*)	Susceptible (*n*)	Sensitivity (Interpreted), % (95% CI)	Specificity (Interpreted), % (95% CI)	Cohen’s Kappa (Interpreted), (95% CI)
*rpoB*					
Mutated	41	1 ^1^	97.6 (97.6)	100.0 (100)	0.95 (0.95)
Wild-type	0	12	(87.4–100)	(73.5–100.0)	(0.85–1.0)
*katG*					
Mutated	34 ^2^	3 ^3^	91.8 (94.3)	100.0 (94.7)	0.88 (0.88)
Wild-type	0	17	(78.0–98.3)	(80.4–100.0)	(0.74–1.0)
pro-*inhA*					
Mutated	14	1 ^4^	93.3 (93.3)	100.0 (100.0)	0.95 (0.95)
Wild-type	0	39	(68.0–99.9)	(90.9–100.0)	(0.86–1.0)

^1^ mutation Val251Phe on *rpoB* gene, not covered by MDR/MTB ELITe MGB^®^. ^2^ including mutation Ser315Gly in an isolate phenotypically susceptible to INH. ^3^ mutations Ser140Gly, Arg249Pro, and Trp689Gly on *katG* gene, not covered by MDR/MTB ELITe MGB^®^. ^4^ mutation −60C > G on *inhA* promoter region, not covered by MDR/MTB ELITe MGB^®^.

## Data Availability

The data presented in this study are available on request from Vichita Ok (vichita.ok@aphp.fr).

## Supplementary Materials

The following are available online at https://www.mdpi.com/article/10.3390/antibiotics10070797/s1, Table S1: Raw Ct values measured by ELITe assay for MTB detection.
