# Supplementary material for: Rapid Molecular Diagnosis of Tuberculosis and Its Resistance to Rifampicin and Isoniazid with Automated MDR/MTB ELITe MGB® Assay
Source: Antibiotics (Basel). 2021 Jun 30;10(7):797. doi: 10.3390/antibiotics10070797 (PMC8300793; doi:10.3390/antibiotics10070797)
Supplement: Supplementary file 1 [file antibiotics-10-00797-s001.zip › antibiotics-1255945-supplementary.pdf]

antibiotics

MDPI

Supplementary Information for

Article

Rapid Molecular Diagnosis of Tuberculosis and Its Resistance to Rifampicin and Isoniazid with Automated MDR/MTB ELITe MGB® Assay

Vichita Ok [1,2], Alexandra Aubry [1,2], Florence Morel [1,2], Isabelle Bonnet [1,2], Jérôme Robert [1,2] and Wladimir Sougakoff [1,2,*]

1 Centre d'Immunologie et des Maladies Infectieuses, Sorbonne Université, INSERM, U1135, Cimi-Paris, F-75013 Paris, France; vichita.ok@aphp.fr (V.O.); alexandra.aubry@sorbonne-universite.fr (A.A.); florence.morel2@aphp.fr (F.M.); isabelle.bonnet2@aphp.fr (I.B.); jerome.robert@aphp.fr (J.R.)

2 AP-HP (Assistance Publique Hôpitaux de Paris), Centre National de Référence des Mycobactéries et de la Résistance des Mycobactéries aux Antituberculeux, Laboratoire de Bactériologie-Hygiène, Groupe Hospitalier Universitaire Sorbonne Université, Hôpital Pitié-Salpêtrière, F-75013 Paris, France

* Correspondence: wladimir.sougakoff@aphp.fr; Tel.: +33-1-42-16-20-97

Table S1. Raw Ct values measured by ELITe assay for MTB detection.

Samples	Ct value	Result
1	> 45	negative
2	> 45	negative
3	> 45	negative
4	26.01	positive
5	35.26	positive
6	> 45	negative
7	> 45	negative
8	36.59	positive
9	26.14	positive
10	29.65	positive
11	26.31	positive
12	25.84	positive
13	31.4	positive
14	> 45	negative
15	> 45	negative
16	> 45	negative
17	22.03	positive
18	> 45	negative
19	> 45	negative
20	> 45	negative
21	> 45	negative
22	> 45	negative
23	> 45	negative
24	> 45	negative
25	> 45	negative
26	> 45	negative
27	21.58	positive
28	27.94	positive
29	21.89	positive
30	27.58	positive

31	> 45	negative
32	28.65	positive
33	> 45	negative
34	> 45	negative
35	> 45	negative
36	> 45	negative
37	> 45	negative
38	> 45	negative
39	> 45	negative
40	> 45	negative
41	> 45	negative
42	24.2	positive
43	> 45	negative
44	> 45	negative
45	> 45	negative
46	> 45	negative
47	> 45	negative
48	> 45	negative
49	> 45	negative
50	32.5	positive
51	> 45	negative
52	30.91	positive
53	> 45	negative
54	> 45	negative
55	30.96	positive
56	24.97	positive
57	> 45	negative
58	> 45	negative
59	> 45	negative
60	> 45	negative
61	> 45	negative
62	> 45	negative
63	> 45	negative
64	37.6	positive
65	> 45	negative
66	> 45	negative
67	36.02	positive
68	> 45	negative
69	> 45	negative
70	> 45	negative
71	> 45	negative
72	> 45	negative
73	> 45	negative
74	> 45	negative
75	> 45	negative
76	> 45	negative
77	37.56	positive
78	> 45	negative
79	39.42	positive
80	> 45	negative
81	> 45	negative
82	39.6	positive
83	> 45	negative
84	> 45	negative

85	> 45	negative
86	> 45	negative
87	> 45	negative
88	26.65	positive
89	> 45	negative
90	> 45	negative
91	> 45	negative
92	> 45	negative
93	> 45	negative
94	> 45	negative
95	30.06	positive
96	35.62	positive
97	> 45	negative
98	> 45	negative
99	> 45	negative
100	37.45	positive
101	> 45	negative
102	> 45	negative
103	> 45	negative
104	> 45	negative
105	> 45	negative
106	35.38	positive
107	26.5	positive
108	> 45	negative
109	34.63	positive
110	> 45	negative
111	> 45	negative
112	> 45	negative
113	> 45	negative
114	> 45	negative
115	> 45	negative
116	> 45	negative
117	33.11	positive
118	> 45	negative
119	> 45	negative
120	> 45	negative
121	> 45	negative
122	> 45	negative
123	> 45	negative
124	> 45	negative
125	> 45	negative
126	> 45	negative
127	> 45	negative
128	> 45	negative
129	> 45	negative
130	> 45	negative
131	> 45	negative
132	> 45	negative
133	24.19	positive
134	33.37	positive
135	> 45	negative
136	24.15	positive
137	> 45	negative
138	> 45	negative

139	> 45	negative
140	> 45	negative
141	> 45	negative
142	37.06	positive
143	> 45	negative
144	> 45	negative
145	> 45	negative
146	> 45	negative
147	> 45	negative
148	> 45	negative
149	> 45	negative
150	> 45	negative
151	> 45	negative
152	> 45	negative
153	> 45	negative
154	> 45	negative
155	36.17	positive
156	35.53	positive
157	29.1	positive
158	> 45	negative
159	> 45	negative
160	> 45	negative
161	> 45	negative
162	> 45	negative
163	> 45	negative
164	> 45	negative
165	> 45	negative
166	29.24	positive
167	> 45	negative
168	> 45	negative
169	> 45	negative
170	> 45	negative
171	> 45	negative
172	> 45	negative
173	> 45	negative
174	> 45	negative
175	34.12	positive
176	> 45	negative
177	> 45	negative
178	> 45	negative
179	> 45	negative
180	> 45	negative
181	> 45	negative
182	> 45	negative
183	33.23	positive
184	22.25	positive
185	> 45	negative
186	> 45	negative
187	> 45	negative
188	> 45	negative
189	> 45	negative
190	> 45	negative
191	> 45	negative
192	> 45	negative

193	> 45	negative
194	> 45	negative
195	> 45	negative
196	> 45	negative
197	> 45	negative
198	> 45	negative
199	> 45	negative
200	> 45	negative
201	> 45	negative
202	> 45	negative
203	> 45	negative
204	> 45	negative
205	> 45	negative
206	> 45	negative
207	> 45	negative
208	> 45	negative
209	> 45	negative
210	> 45	negative
211	> 45	negative
212	> 45	negative
213	> 45	negative
214	> 45	negative
215	> 45	negative
216	> 45	negative
217	33.15	positive
218	40.66	positive
219	36.99	positive
220	> 45	negative
221	37.56	positive
222	> 45	negative
223	> 45	negative
224	> 45	negative
225	> 45	negative
226	> 45	negative
227	> 45	negative
228	> 45	negative
229	> 45	negative
230	> 45	negative
231	> 45	negative
232	> 45	negative
233	30.73	positive
234	> 45	negative
235	> 45	negative
236	> 45	negative
237	21.42	positive
